# Quantitative evaluation of neuroradiological and morphometric alteration of inferior Fronto-Occipital Fascicle across different brain tumor histotype: an Italian multicentric study

**DOI:** 10.1007/s00701-025-06488-6

**Published:** 2025-03-12

**Authors:** Roberto Altieri, Andrea Bianconi, Stefano Caneva, Giovanni Cirillo, Fabio Cofano, Sergio Corvino, Oreste de Divitiis, Giuseppe Maria Della Pepa, Ciro De Luca, Pietro Fiaschi, Gianluca Galieri, Diego Garbossa, Giuseppe La Rocca, Salvatore Marino, Edoardo Mazzucchi, Grazia Menna, Antonio Mezzogiorno, Alberto Morello, Alessandro Olivi, Michele Papa, Daniela Pacella, Rosellina Russo, Giovanni Sabatino, Giovanna Sepe, Assunta Virtuoso, Giovanni Vitale, Rocco Vitale, Gianluigi Zona, Manlio Barbarisi

**Affiliations:** 1 Multidisciplinary Department of Medical-Surgical and Dental Specialties, University of Campania “Luigi Vanvitelli”, 80131 Naples, Italy; 2 Department of Neuroscience, Rehabilitation, Ophthalmology, Genetics, Maternal and Child Health (DINOGMI), University of Genova, 16132 Genova, Italy; 3 Department of Neurosurgery, IRCCS Ospedale Policlinico San Martino, Genova, 16132 Genoa, Italy; 4 Laboratory of Morphology of Neuronal Network, Department of Public Medicine, University of Campania “Luigi Vanvitelli”, Naples, Italy; 5 Neurosurgery Unit, Department of Neuroscience “Rita Levi Montalcini”, University of Turin, Via Cherasco, 15, 10126 Turin, Italy; 6 Department of Neuroscience and Reproductive and Odontostomatological Sciences, Neurosurgical Clinic, School of Medicine, University of Naples “Federico II”, Via Pansini, 5, 80131 Naples, Italy; 7 Institute of Neurosurgery, Fondazione Policlinico Universitario A. Gemelli IRCCS, Catholic University, 00168 Rome, Italy; 8 Neurosurgical Training Center and Brain Research, Mater Olbia Hospital, 07026 Olbia, Italy; 9 Department of Neurosurgery, IRCCS Regina Elena National Cancer Institute, 00144 Rome, Italy; 10 Department of Public Health, University Federico II, Naples, Italy; 11 Department of Radiology, Neuroradiology Unit, Fondazione Policlinico Universitario A. Gemelli IRCCS, Rome, Italy; 12 Neurosurgery Unit, Regional Hospital San Carlo, Potenza, Italy; 13 Division of Neurosurgery, “Ospedale del Mare” Hospital, Naples, Italy

**Keywords:** Glioblastoma, Low Grade Glioma, Metastasis, Meningioma, IFOF, Connectome

## Abstract

**Background:**

Inferior Fronto-Occipital Fascicle (IFOF) is a multitasking connection bundle essential for communication and high level mentalization. The aim of the present study was to quantitatively assess its radiological-anatomical-morphometric modifications according to different brain tumor histotype.

**Methods:**

A retrospective multicentric Italian study was conducted. IFOF reconstructions were calculated for both hemispheres for each patient diagnosed with Glioblastoma (GBM), Low Grade Glioma (LGG), Brain Metastasis and Meningioma using Elements Fibertracking software (Brainlab AG). A 3D object of each fascicle was evaluated for volume, average fractional anisotropy (FA) and length. The cerebral healthy hemisphere was compared to the pathological contralateral in different tumor histotype.

**Results:**

1294 patients were evaluated. 156 met the inclusion criteria. We found a significant difference between healthy hemisphere and the contralateral for IFOF mean length and volume (*p*-value < 0.001). Considering GBM subgroup, Student’s t-test confirmed the results. In LGG subgroup, there was significant difference between the 2 hemispheres for IFOF mean length, mean FA and volume (respectively *p*-value 0.011; *p*-value 0.021, *p*-value < 0.001). In patients affected by brain metastasis (18) Student’s t-test showed a significant difference for FA and volume (*p*-value 0.003 and 0.02 respectively). No differences were found in patients affected by meningiomas.

**Conclusions:**

The careful preoperative neuroradiological evaluation of the brain-tumor interface is indispensable to plan a tailored surgical strategy and perform a safe and effective surgical technique. It depends on the tumor histology and pattern of growth. GBM have a mixed component, with the solid enhancing nodule which accounts for IFOF displacement and the peritumoral area which accounts for an infiltrative/destructive effect on the fascicle. LGG determine a prevalent infiltrative pattern. Metastases determine an IFOF dislocation due to peritumoral oedema. Meningiomas do not impact on WM anatomy.

## Introduction

**Fig. 1:**
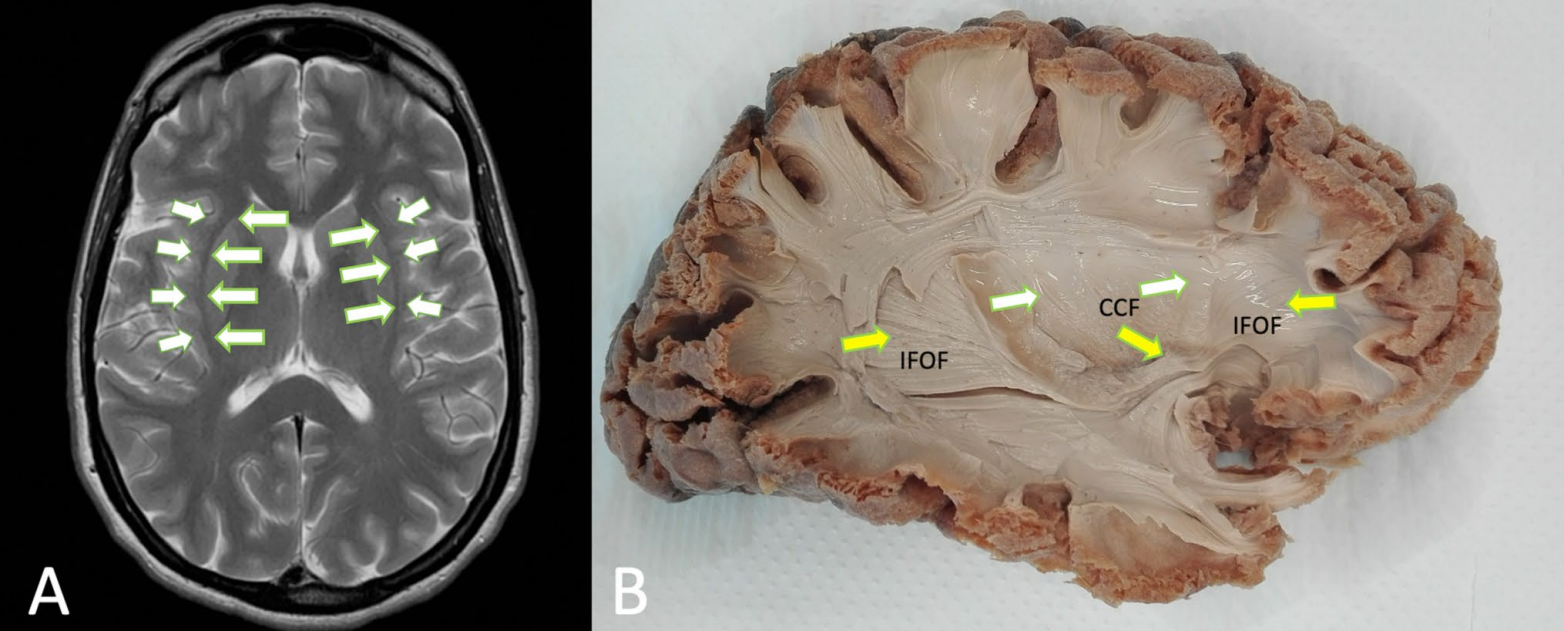
**A **brain MRI T2 axial section highlighting the external capsule (arrows); **B **lateral view of the external capsule in brain specimen, showing claustro-cortical fibers (CCF) (white arrows) and IFOF (yellow arrows). The white matter dissection was carried out in the anatomy lab on a human adult brain embalmed with the Klingler technique. The dissection was performed removing the cortex with a blunt spatula and then dissecting the fibers from lateral to medial.”

In recent decades, advances in technology and therapies leading to increased life expectancy have driven a paradigm shift in neuro-oncological surgery. The focus has moved from prioritizing maximal tumor resection to emphasizing the preservation of the patient’s neurological function and quality of life. This approach sometimes favors subtotal resection followed by adjuvant therapies or multiple re-operations, striving to achieve an optimal balance between oncological control and functional outcomes [28]. For this purpose, a thorough understanding of the brain’s neurofunctional microsurgical anatomy, including the location of eloquent cortical areas and the pathways of subcortical white matter (WM) fiber tracts, is essential for safe and effective neurosurgery. In this setting, the emerging concept of “connectome” has transformed the perspective of neuro-oncological surgery, emphasizing the importance of preserving the brains intricate network of connections [6, 12, 33]. The onco-functional balance now hinges on the ability to remove the tumor while preserving the integrity of key white matter (WM) tracts [1, 10, 24]. Consequently, the optimal surgical strategy requires a clear understanding of the anatomical relationship between the tumos growth pattern and the surrounding WM [18, 25]. In this context, the Inferior Fronto-Occipital Fasciculus (IFOF), stands out among the various association fibers as a major white matter tract, is a large white matter tract running from the occipito-parietal region to the frontal lobe, coursing below to the insula and composing the inferior third of external capsules (Fig. 1). The IFOF serves as a versatile connection within each hemisphere and plays a pivotal role in functions such as communication and advanced mentalization [4, 44].

Complexity of central nervous system (CNS) and higher functions, including human behavior, derives from the intrinsic organization of the brain into anatomically and functionally well-defined neural circuits. Every change of the brain anatomy can potentially subvert brain function. Recently the different and intricate functional interplay between brain tumors and the brain cellular microenvironment, encompassing neurons, glia, and vessels has been reported [2, 17, 31, 35, 39, 46]. Recently Prof Corbetta et Al. underline the role of connections contribute to the location, spread, and recurrence of glioblastoma (GBM)[5]. However, the relationship between changes in subcortical connections and tumors with different growth patterns remains not fully understood [20]. Although GBM is still considered an infiltrative and diffuse brain disease, clinical and surgical experience suggests a growth pattern that primarily displaces and pushes fiber bundles [18, 22, 23]. As a result, patients signs and symptoms are usually due to the mass effect before surgery, being not suitable candidates for awake surgery. Moreover, this growth pattern sometimes allows for the identification of a pseudo-plane, facilitating the disconnection of the tumor from the surrounding functional brain [43]. In this scenario, we aim to quantitatively assess radiological, anatomical and morphometric changes of the IFOF across several brain tumor histotype, with different histology, biological behavior, and growth patterns.

## Materials and methods

### Study design and patients

A retrospective multicentric Italian study involving 2 Neurosurgical Units of the north of Italy (University of Turin and I.R.C.C.S. Ospedale Policlinico San Martino, Genoa), 2 Neurosurgical Units of the center of Italy (Università Cattolica del Sacro Cuore, Rome, and Neurosurgical Unit of Mater Olbia Hospital, Olbia) and 2 Neurosurgical Units of the south of Italy (University of Campania "Luigi Vanvitelli", Naples and A.O.R. San Carlo, Potenza) was made.

Medical record data of 1294 patients operated on for intracranial glioblastoma, low-grade glioma, metastasis and meningioma, between January 2023 and December 2023, have been retrospectively reviewed. Inclusion criteria were: i) patient’s age > 18 years old, ii) histological and/or molecular diagnosis of GBM, LGG, brain metastasis and meningioma, iii) complete preoperative neuroradiological studies, including contrast-enhanced magnetic resonance imaging (MRI) with sequences T1-weighted, Fluid-Attenuated-Inversion-Recovery (FLAIR) and Diffusion Tensor Imaging (DTI), iv) tumors in close proximity to the IFOF course. Patients were classified in four groups according to the tumor hosted: A) GBM; B) LGG; C) metastasis; D) meningioma.

Despite other white matter fibers tracts, especially superior longitudinal fascicle (SLF), could be relevant for the study, our choice to focus on the IFOF was due to the anatomical distribution of our surgical series; most patients harbored brain tumors located in proximity of the course of the IFOF, mainly because of the natural wider anatomical distribution of this fascicle, and to avoid bias, such as heterogeneity of the data considering more than one WM fibers tract, we preferred to use only IFOF. However, SLF and Arcuate Fascicle could be studied in future projects.

### MRI protocol and analysis

Imaging examinations were performed using a 1.5 Tesla MRI with conventional sequences at A.O.R. San Carlo and a 3 Tesla MRI at the Academic Institutes. DTI acquisitions were done with 32 directions at University of Turin, A.O.R. San Carlo, University of Campania "Luigi Vanvitelli" and Mater Olbia Hospital while they were done with 60 directions at I.R.C.C.S. Ospedale Policlinico San Martino and 64 at Università Cattolica del Sacro Cuore.

Pre-operative fiber tracking reconstruction was evaluated. IFOF reconstructions were calculated for both hemisphere for each patient by an expert team of neurosurgeons, neuroradiologist and neuroanatomists using Elements Fibertracking and SmartBrush software (Brainlab AG). Region of interest (ROI) were positioned as previously reported by Feconja et al.[26]. In detail, were manually placed in the frontal lobe, specifically in the subcallosal and ventral premotor cortices, and the occipital lobe, including the lateral occipital cortex. Using deterministic tractography, we generated the IFOF pathways by seeding from the ROIs and following the streamline trajectories that connect these regions. Tractography parameters were set to include streamlines with a minimum length of 50 mm and an FA threshold of 0.15. Additionally, streamlines passing through the extreme capsule were included, while those deviating into adjacent white matter tracts were excluded. Tractography results were normalizing using a standard anatomical template to minimize individual differences. This step ensured that the IFOF pathways were consistently aligned across all participants, effectively reducing inter-individual variability in the tract reconstructions.

A 3D object of each fascicle from both healthy and contralateral side was evaluated for volume, average fractional anisotropy (FA) and length. For our study, we used an FA threshold of 0.15 and a maximum angle setting of 60 degrees. These settings were chosen based on the default recommendations provided by the Elements software.

Finally, the healthy cerebral hemisphere was compared with the pathological contralateral one.

### Statistical analysis

Data were reported as mean values and standard deviation for quantitative variables and with absolute frequencies and percentages for categorical variables. Differences between the means of the variables for the affected and healthy sides were computed with Student’s t-test for paired samples. Analysis of the determinants of the paired mean differences between the healthy and affected sides was conducted with multiple mixed-effect linear regression, considering patient’s ID as random effect. For all analyses, a two-tailed p < 0.05 was considered significant. All analyses are performed using the statistical software R version 4.4.0.

## Results

In the selected period, 1294 patients underwent neuro-oncological surgery at considered Institutions. Among these patients, 156 met the inclusion criteria. 98 (63%) were affected by GBM, 26 (17%) by a Low-Grade Glioma (LGG), 14 (9.0%) had a Meningioma and 18 (12%) had a brain metastasis.

83 patients were males (53%) and 73 were females (47%). 109 (70%) patients had a tumor located into the left cerebral hemisphere while the remaining 47 (30%) had a tumor on the contralateral side. Mean age was 59.

All demographic data are summarized in Table 1.

**Table 1:** Demographic and clinical data

Covariates	*N* = 156
Sex	
Female	73 (47%)
Male	83 (53%)
Age (y)	59 (13)
Diagnosis	
GBM	98 (63%)
LGG	26 (17%)
Meningioma	14 (9.0%)
Metastasis	18 (12%)
Location	
Frontal (bilaterally)	1 (0.6%)
Clinoid	1 (0.6%)
Frontal	45 (29%)
Frontal insular	2 (1.3%)
Fronto-parietal	7 (4.5%)
Fronto-temporal	4 (2.6%)
Fronto-temporo-insular	6 (3.8%)
Insular	1 (0.6%)
Occipital	1 (0.6%)
Parasagittal	1 (0.6%)
Parietal	16 (10%)
Parieto-occipital	8 (5.1%)
Parieto-temporal	4 (2.6%)
Temporal	44 (28%)
Temporal-parietal	9 (5.8%)
Temporo-insular	4 (2.6%)
Temporo-occipital	1 (0.6%)
Thalamic	1 (0.6%)
Side	
Left	109 (70%)
Right	47 (30%)
Volume	
Enhancing nodule (EN) cm^3^	25 (21)
FLAIR hyperintensity (beyond EN) cm^3^	49 (34)

*GBM *glioblastoma multiforme, *LGG* low-grade gliomas, *IFOF* inferior fronto-occipital fasciculus, *FA* fractional anisotropy. Data presented as mean (SD) or frequency (%)

The mean volume of enhancing nodule (EN) was 25 cm3 while the mean FLAIR volume beyond EN was 49 cm3.

The mean length of the IFOF of healthy side and contralateral side was 118 mm and 112 mm, respectively. The mean FA of healthy side and contralateral side was 0.51 and 0.42, respectively. The mean IFOF volume of healthy side and contralateral side was 39 cm^3^ and 28 cm^3^ respectively.

All these quantitative measures of IFOF in healthy and affected side are summarized in Table 2.

**Table 2:** Quantitative measures of IFOF in healthy and affected side

IFOF	Healthy side	Affected side
Mean length mm (SD)	118 (22)	112 (24)
FA	0.51 (0.85)	0.42 (0.05)
Volume cm^3^ (SD)	39 (12)	28 (11)

*GBM* glioblastoma multiforme, *LGG* low-grade gliomas, *IFOF* inferior fronto-occipital fasciculus, *FA* fractional anisotropy. Data presented as mean (SD) or frequency (%)

Considering overall sample (156 pt) Student’s t-test showed a significant difference between healthy hemisphere and hemisphere harboring tumor for IFOF mean length and its volume (*p*-value < 0.001). Considering only patients with GBM (98) Student’s t-test confirmed the results. Student’s t-test, evaluating LGG patients (26), showed a significant difference between healthy hemisphere and the hemisphere harboring tumor for IFOF mean length, mean FA and volume (respectively *p*-value 0.011; *p*-value 0.021, *p*-value < 0.001). In patients affected by brain metastasis (18) Student’s t-test showed a significant difference for FA and volume (*p*-value 0.003 and 0.02 respectively). No differences were found in patients affected by meningiomas (14).

### Correlation analysis

Hemisphere status, healthy vs affected side, was significantly associated with IFOF mean length; in particular, healthy side had a significantly higher IFOF mean length than hemisphere harboring tumor (adj. β = 5.7, 95% CI 4.2, 7.3, *p* =  < 0.001). Tumor histology was significantly associated with IFOF mean length: in particular, LGG had a significantly lower IFOF mean length than GBM (adj. β = −12, 95% CI −23, −0.64, *p* = 0.039) while meningioma had an IFOF mean length significantly higher (adj. β = 37, 95% CI 26, 48, p < 0.001). EN volume also was significantly associated with IFOF mean length (adj. β = −0.40, 95% CI −0.59, −0.21, *p* =  < 0.001).

Hemisphere status was also significantly associated with IFOF volume; healthy side had a significantly higher IFOF volume than contralateral (adj. β = 10, 95% CI 8.4, 12, *p* =  < 0.001).

Gender patient was significantly associated with IFOF volume; male had a significantly higher IFOF volume than female (adj. β = 3.1, 95% CI 0.50, 5.8, *p* =  < 0.02).

Tumor histology was significantly associated with IFOF volume: LGG had a significantly higher IFOF volume than GBM (adj. β = 8.9, 95% CI 3.8, 14, *p* =  < 0.001) while meningioma had a significantly lower IFOF volume (adj. β = −7.7, 95% CI −12, −3.0, *p* = 0.002).

EN volume and FLAIR volume were both significantly associated with IFOF volume (respectively adj. β = 0.15, 95% CI 0.07, 0.24, *p* =  < 0.001 and adj. β = −0.10, 95% CI −0.15, −0.06, *p* =  < 0.001) (Table 3).

**Table 3:** Analysis of the determinants of the paired mean differences between the healthy and affected brain hemispheric sides

	Outcome: IFOF mean length (mm)			Outcome: IFOF volume (cm^3^)		
Characteristic	Adj. β	95% CI	*p*-value	Adj. β	95% CI	*p*-value
Group						
Affected	—	—		—	—	
Healthy	5.7	4.2, 7.3	** < **0.001	10	8.4, 12	** < **0.001
Sex						
Female	—	—		—	—	
Male	0.62	−5.4, 6.6	0.838	3.1	0.50, 5.8	0.020
Age (y)	0.01	−0.28, 0.30	0.939	−0.08	−0.20, 0.05	0.233
Diagnosis						
GBM	—	—		—	—	
LGG	−12	−23, −0.64	0.039	8.9	3.8, 14	** < **0.001
Meningioma	37	26, 48	** < **0.001	−7.7	−12, −3.0	0.002
Metastasis	6.4	−3.2, 16	0.187	−0.73	−5.0, 3.5	0.732
Side						
Left	—	—		—	—	
Right	0.08	−6.5, 6.7	0.980	−0.33	−3.2, 2.6	0.820
Volume – EN cm^3^	−0.40	−0.59, −0.21	** < **0.001	0.15	0.07, 0.24	** < **0.001
Volume—FLAIR hyperintensity beyond EN cm^3^	0.08	−0.02, 0.18	0.131	−0.10	−0.15, −0.06	** < **0.001

*IFOF*, inferior fronto-occipital fasciculus, *Adj. β* adjusted beta value, *CI* confidence interval, *GBM* glioblastoma multiforme, *LGG* low-grade gliomas, *EN* enhancing nodule

The multiple mixed-effect linear regression conducted on the subgroup of GBM showed that healthy side had a significantly higher IFOF mean length and volume than contralateral (respectively adj. β = 7.4, 95% CI 5.5, 9.3, *p* =  < 0.001 and adj. β = 12, 95% CI 9.5, 14, *p* =  < 0.001). In these subgroups it was evidenced that EN volume was significantly associated with IFOF mean length and IFOF volume (respectively adj. β = −0.51, 95% CI −0.74, −0.27, *p* =  < 0.001 and adj. β = 0.19, 95% CI 0.09, 0.29, *p* =  < 0.001). IFOF volume was moreover significantly associated, in GBM subgroup with, FLAIR volume of areas beyond EN (adj. β = −0.09, 95% CI −0.14, −0.03, *p* = 0.002) (Table 4).

**Table 4:** Analysis of the determinants of the paired mean differences between the healthy and affected brain hemispheric sides in the GBM subgroup

	Outcome: IFOF mean length (mm) in GBM			Outcome: IFOF volume (cm^3^) in GBM		
Characteristic	Adj. β	95% CI	*p*-value	Adj. β	95% CI	*p*-value
Group						
Affected	—	—		—	—	
Healthy	7.4	5.5, 9.3	** < **0.001	12	9.5, 14	** < **0.001
Sex						
Female	—	—		—	—	
Male	2.9	−5.0, 11	0.469	3.2	−0.15, 6.5	0.061
Age (y)	0.16	−0.23, 0.56	0.415	−0.13	−0.30, 0.04	0.132
Side						
Left	—	—		—	—	
Right	−1.4	−10, 7.3	0.751	−0.05	−3.7, 3.6	0.977
Volume – EN cm^3^	−0.51	−0.74, −0.27	** < **0.001	0.19	0.09, 0.29	** < **0.001
Volume—FLAIR hyperintensity beyond EN cm^3^	−0.02	−0.15, 0.11	0.752	−0.09	−0.14, −0.03	0.002

*IFOF*, inferior fronto-occipital fasciculus, *Adj. β* adjusted beta value, *CI* confidence interval, *GBM* glioblastoma multiforme, *EN* enhancing nodule

## Discussion

Brain tumors present diverse challenges to neurosurgeons, as their histology, biological behavior, and growth patterns vary widely, influencing their interaction with surrounding brain structures, particularly WM fibers. GBM, diffuse LGG, metastases, and meningiomas each exhibit distinct growth characteristics that impact both the planning and execution of surgical resection. Understanding the specific anatomical and pathological features of these tumors is essential for optimal surgical outcomes, as it enables the surgeon to differentiate between tumor and healthy tissue, identify safe dissection planes, and preserve critical brain functions. This introduction highlights the unique aspects of these tumor types and the importance of detailed preoperative planning in guiding safe and effective surgical intervention.

In this setting it is worthy to remember that there is not unanimous consent in literature about the IFOF’s asymmetry in healthy human brain. A rightward asymmetry of IFOF for tract streamlines, but not tract volume, was suggested in a study of 40 right-handed healthy brain [38] and confirmed in a following study including 60 healthy patients [45]. However, this result has not been confirmed in following studies [14, 41]. Wu et al. have investigated the connectivity, asymmetry and segmentation of the left and right IFOF in 10 healthy adults using high angular diffusion spectrum imaging (DSI) analysis and a 90-subject DSI template using HARDI tractography. The statistical results revealed no asymmetry between the left and right hemispheres and no significant differences existed in distributions of the IFOF according to sex.

A matter of debate is also the asymmetry of the IFOF’s layers: while Hau et al.[45] suggested that the ventral layer may have rightward asymmetry and the dorsal leftward asymmetry in volume, on the other hand Vassal et al. suggested opposite asymmetry100 and asymmetrical patterns were not replicated by others [14, 41].

Results from the present study showed that brain tumors significantly affect the mean length and volume of the IFOF. The healthy brain hemisphere exhibits greater IFOF length and volume compared to the contralateral side. Males have a higher IFOF volume probably due to physiological phenotypical differences gender related.

**Fig. 2:**
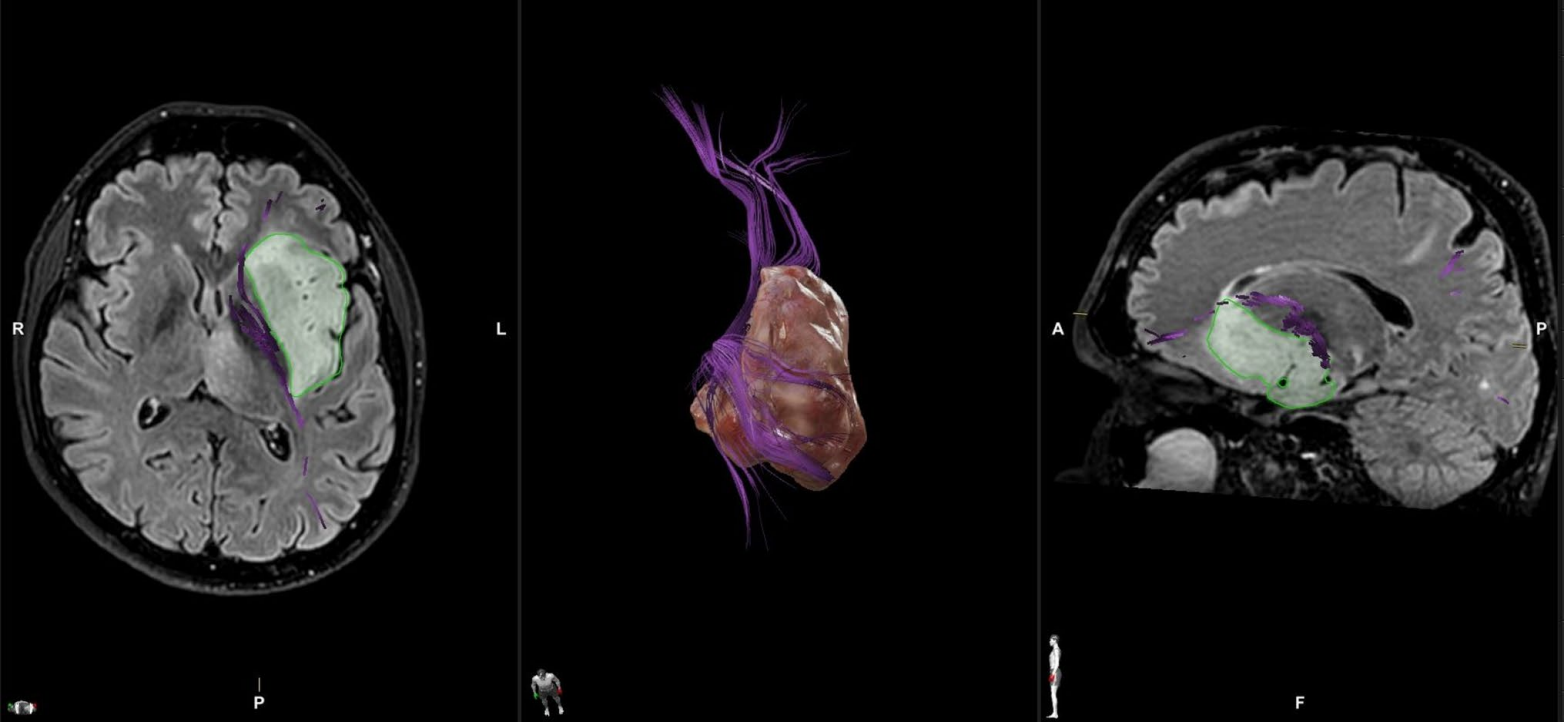
Insular GBM determining a dislocation and a reduction of IFOF volume and mean length

Considering histological subgroups, our findings highlight that GBM causes substantial changes in the structural anatomy of the IFOF, affecting both its mean length and volume. These alterations can be attributed to a combination of destruction and displacement of the bundle due to the rapid growth of the tumor [18] (Fig. 2). GBM is a high-grade, diffusely infiltrating glioma marked by nuclear atypia, high mitotic activity, microvascular proliferation, and necrosis. It primarily affects subcortical WM and spreads to adjacent structures along WM fibers, often displaying "secondary structures" like perineuronal satellitosis and perivascular aggregates. The tumor typically exhibits a bulky growth pattern with a compressive effect on nearby WM. Proper identification of anatomical tumor boundaries is crucial for neurosurgeons to safely disconnect the tumor from the surrounding WM, allowing resection along a safer plane [22, 43].

**Fig. 3:**
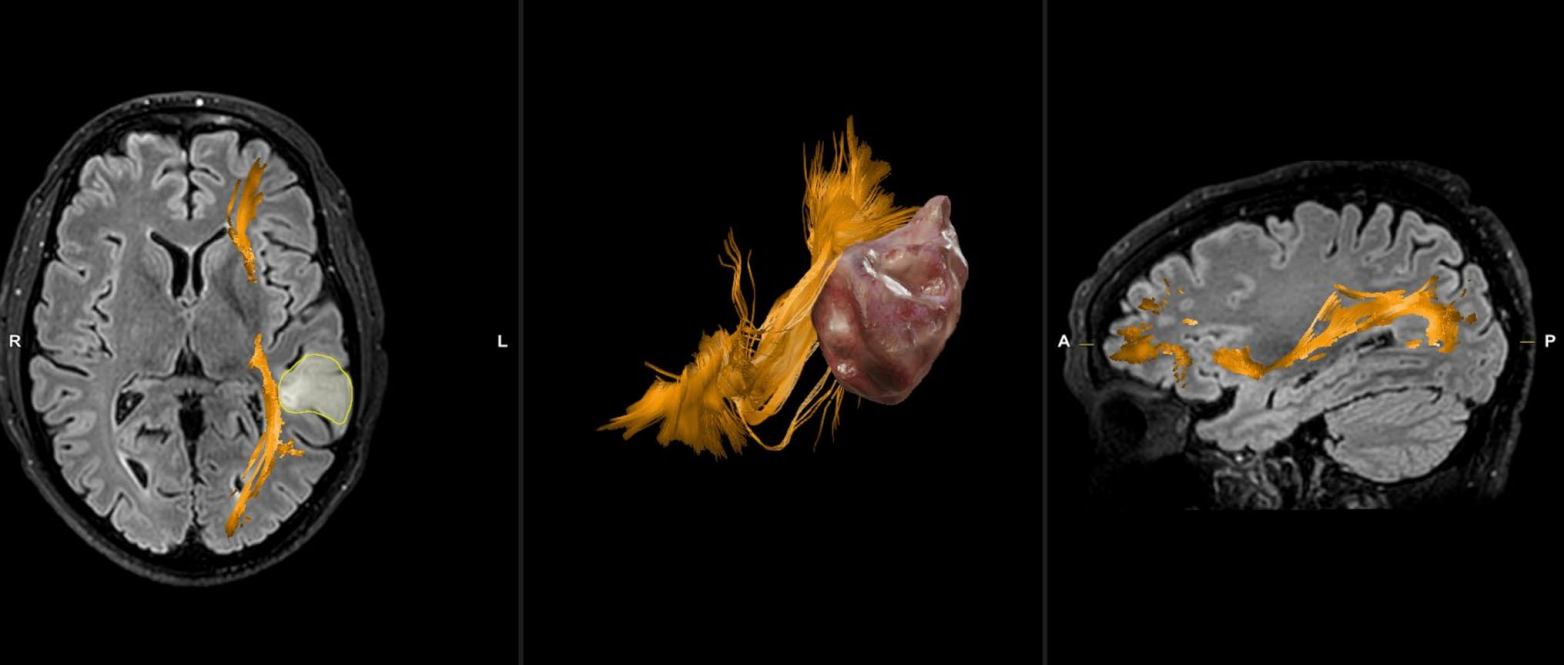
Higher IFOF volume in parietal LGG compared to GBM

In contrast, LGGs primarily impact the IFOF mean length, FA, and volume. LGGs have a shorter mean length but a larger volume compared to GBM, likely due to the infiltrative nature of these slow-growing tumors and the development of compensatory neuroplasticity (Fig. 3). Despite the extent of resection represents one of the main prognostic factors of progression-free and overall survival, considering the slow growth of LGG, with overall survival estimated over 10 years, the current surgical approach recommends the prevention of new permanent neurological deficits more important than the extent of resection. For this purpose, a meticulous preoperative planning, assisted by neuropsychological and neuroradiological examinations, as well as intraoperative tools, are mandatory. LGG are characterized by glial cells that diffusely infiltrate the brain parenchyma individually, usually without cellular cohesion, enlarging and distorting the invaded anatomical structures, mainly the adjacent white matter fibers. The term “diffuse” refers to the prevalent infiltrative component of these gliomas. However, despite the absence of a bulky growth pattern, the LGG boundaries also are anatomically defined by the sulci/gyri. Therefore, in this case also, the circumferential sulcus-guided technique could improve the possibility to achieve a supramarginal resection [36]. However, it is more difficult differentiate the normal WM from tumor and it is mandatory identify preoperatively the anatomical relationship between healthy brain and LGG.

Brain metastasis impact on IFOF FA and volume because the bundles are only dislocated by the peritumoral oedema. The main treatment strategies for brain metastases are surgery and radiosurgery, with success depending on the real correspondence between the focality of disease at macroscopic and microscopic extent of disease. In this setting, the brain/metastasis interface may assume a relevant significance. Metastases of cerebral hemispheres are usually located in arterial border zones and at the junction of the cerebral cortex and white matter [29]. They may exhibit 3 distinct patterns of growth: well-demarcated growth, vascular co-option, and diffuse infiltration (pseudogliomatous growth) in the adjacent parenchyma. Most brain metastases are fairly well demarcated, circumscribed by a rim of gliotic tissue that separates the tumor from the surrounding healthy brain tissue which results displaced, providing a safe dissection plane of resection which allows the goal of a gross total resection through the advisable en-bloc technique with circumferential dissection [19, 30]. Nevertheless, due to their intra-axial localization, superficial or deep, a certain degree of cortex and subcortical WM must necessarily be traversed.

In our series, despite the peritumoral edema close to the IFOF course, meningiomas, do not cause modifications to IFOF anatomy and have a significantly higher IFOF mean length than GBM, owing to their extra-axial growth. Surgery represents the gold standard of treatment for symptomatic meningiomas in patients in good clinical condition, with the aim of maximal safe tumor resection while preserving the neurological function. One of the main factors affecting the surgical strategy is the brain-tumor interface which depends on the tumor pattern of growth. Meningiomas are extra-axial dural-based tumors arising from the arachnoid cap-cells and in most cases (80%) are benign (WHO grade 1) and without invasion of the brain parenchyma. Indeed, as extrinsic tumors arising outside the brain, meningiomas just buckle the cortex inward, displacing rather disrupting or infiltrating into adjacent WM tracts [15]; their pattern of growth provides a discernible CSF-vascular cleft (well delineated on T2-weighted sequences) between the tumor and the underlying cortex, representing a clear arachnoid surgical plane of cleavage which in turn allows tumor resection without injury of the cortical and subcortical structures following the 4D-rule proposed by Fukushima. Sometimes, however, this plane is not present due to tumor-pial adhesion or cortical invasion like in WHO grade 2 or 3 meningiomas. In these cases, mainly for lesions in eloquent areas like Rolandic meningiomas, including parasagittal and falx meningiomas involving the Rolandic area, some authors have proposed the use of intraoperative monitoring to decrease the risk of iatrogenic postoperative functional deficit [40].

In our study, the volume of the EN is associated with a shorter IFOF mean length and a larger IFOF volume, while FLAIR volume correlates with a smaller IFOF volume. These findings can be explained by the bulky effect of the EN, which causes WM displacement, while the infiltrative nature of FLAIR results in fascicle destruction [9, 16, 37]. In the GBM subgroup, EN volume leads to a reduction in IFOF mean length and an increase in IFOF volume, suggesting the pushing effect of the central core nodule on the WM bundle. In contrast, FLAIR volume is associated with a decrease in both IFOF mean length and volume, further confirming the infiltrative nature of the peritumoral areas. In the future, density-based DTI (as opposed to FA-based) with quantitative anisotropy may further demonstrate the distinct pattern of invasiveness and disruption of white matter anatomy by various disease sets [27].

### The role of subcortical WM in tumor growth patterns: insights from advanced neuroimaging

Despite the critical role of subcortical connections in human brain function, their anatomy remains less familiar to many neurosurgeons. However, a thorough understanding of subcortical WM should be a cornerstone of daily surgical practice, as it is well established that WM is more vulnerable to injury and has limited plasticity or recovery potential compared to the eloquent cortex [42].

Advanced neuroimaging techniques, such as fiber tractography, can aid in identifying subcortical bundles during preoperative planning and can be integrated into the stealth navigation system to assist neurosurgeons during tumor resection. Bello et al. showed the concordance between DTI and DES in retrospective series of 64 patients affected by gliomas and found that DTI has a 97% sensitivity for naming sites [8]. DTI is an MRI technique that can indirectly evaluate the integrity of the WM by measuring water diffusion and its directionality [21]. DTI parameters, such as FA, have been used to pre operatively assess WM infiltration [11]. In other hands DTI is a non-invasive technique that give us the possibility to reconstruct the WM bundles thanks to the orientation and location of anisotropic diffusion of hydrogen molecules of water. I t is also helpful, to evaluate the different relation between glioma and the adjacent WM (fiber dislocation, infiltration and destruction) analyzing the FA. Lu et al. [34] hypothesized that in all intracranial neoplasms, increases in peritumoral mean diffusivity are determined primarily based on the free extracellular water content, with corresponding decreases in FA as the extracellular space enlarges. However, in gliomas, mechanisms such as WM disruption or displacement led to further decreases in FA. Indeed these authors suggested that lower FA values in the GBM peritumoral areas are a consequence of the destructive effect of tumour-derived cells on WM fibers [34]. Camins et al. [3] for example, evaluated the pattern of IFOF dislocation in 34 patients affected by gliomas using FA while Ius et Al. studied SLF and IFOF FA in 26 LGG to predict the functional outcome after resection [13].

Finally, it is worthy to remember that along with surgical resection, white matter fibers of the brain are also highly vulnerable to radiation, which lead to acute, subacute and tardive changes ranging from demyelination and mild axonal degradation to radiation necrosis and which extend beyond the target volume of therapy [7]. In addition, chemotherapic agents for gliomas can lead to significant alterations in white matter fibers within the brain, resulting in cognitive deficits [32]. All these structural changes can be detected through DTI [7].

### Significance of the study

By identifying distinct patterns of impact, this study provides insights into the pathophysiological mechanisms associated with different tumor histotype. These findings can enhance the accuracy of preoperative planning, inform targeted therapeutic strategies, and support prognostic assessments by correlating white matter alterations with tumor characteristics.

### Limitations of the study

Our work has some limitations worth noting. The images were acquired using different MRI machines and various DTI direction acquisitions across different institutions; “the variation in magnetic field strengths (1.5 T vs. 3 T), as well as the variation in the number of DTI directions (MPG axes) across different MRI scanners, are notable limitation in tractography studies”.

However, the reconstructions were carried out using consistent methods by a specialized team of neuroanatomists, neuroradiologists, and neurosurgeons, which helps to mitigate these limitations. The heterogeneous tumor types were included to compare the distinct growth patterns of each histotype and their impact on surrounding white matter bundles. Unfortunately, the size of some subgroups is limited due to clinical priorities, as neuroradiologists often allocate more time to DTI for intra-axial tumors located in eloquent areas. While the variability in tumor locations and the absence of clinical outcomes may be seen as limitations, the primary focus of our study is the radiological evaluation of the IFOF in relation to different tumor histotype, independent of clinical features. Given that the IFOF is a long association bundle spanning the entire hemisphere, every tumor location affects at least part of this fascicle. The IFOF along with various cognitive functions, such as language, attention, vision, may also be involved in the handedness and laterality; however, we did not consider this aspect in our study.

## Conclusion

Brain anatomy is intricately linked to function, and any brain pathology can lead to dysfunction. Different brain tumor histotypes exhibit distinct histology, biological behavior, and growth patterns, which result in varying microscopic interactions with WM fibers. Understanding these relationships from both pathological and neuroradiological perspectives is essential for ensuring safe surgical outcomes. The IFOF is a multifunctional WM bundle and a key component of several critical brain networks. In this study, despite limitations, we have described its anatomical changes in relation to the growth patterns of various brain tumors. GBM exhibits a mixed impact, with displacement of the IFOF due to EN volume and an infiltrative/destructive effect from FLAIR peritumoral areas. LGG, on the other hand, shows a predominantly infiltrative growth pattern with compensatory neuroplasticity. Metastases, being "extra-axial" tumors, lead to IFOF displacement due to peritumoral edema. Meningiomas, however, do not affect WM anatomy. We believe these findings could be valuable in preoperative planning to tailor the surgical approach for each patient.

## Data Availability

No datasets were generated or analysed during the current study.

## Abbreviations

GBM: Glioblastoma
LGG: Low-Grade Glioma
MRI: Magnetic Resonance Imaging
CT: Computed Tomography
WM: White Matter
IFOF: Inferior Fronto-Occipital Fasciculus
CNS: Central Nervous System
FLAIR: Fluid-Attenuated-Inversion-Recovery
DTI: Diffusion Tensor Imaging
EN: Enhancing Nodule
FA: Fractional Anisotropy
SLF: Superior Longitudinal Fasciculus
